# Editorial: Sub- and Supra-Second Timing: Brain, Learning and Development

**DOI:** 10.3389/fpsyg.2016.00747

**Published:** 2016-05-18

**Authors:** Lihan Chen, Yan Bao, Marc Wittmann

**Affiliations:** ^1^Department of Psychology and Beijing Key Laboratory of Behavior and Mental Health, Peking UniversityPeking, China; ^2^Key Laboratory of Machine Perception (Ministry of Education), Peking UniversityBeijing, China; ^3^Human Science Center, Institute of Medical Psychology, Ludwig Maximilian University MunichMunich, Germany; ^4^Institute for Frontier Areas of Psychology and Mental HealthFreiburg, Germany

**Keywords:** subjective time, time perception, time coordination, movement timing, timing mechanisms

Time perception in the range of milliseconds to a few seconds is essential for many important sensory and perceptual tasks including speech perception, motion perception, motor coordination, and cross-modal interaction. For the brain to be in synchrony with the environment, the physical differences in the speeds of light and sound, as well as stimuli from other modalities such as odors, must be processed and coordinated (Pöppel and Bao, 2014; Bao et al., 2015).

Time is a subjective feeling that is modulated by emotional states which trigger temporal distortions (temporal dilation vs. contraction; Wittmann, 2016), hence give rise to subjective time that may be different to *event time* as initially registered in the brain. Recent research suggests that time perception in a multisensory world is subject to prior task experience and shaped by (statistical) learning processes. Humans are active learners. That is, the engagement of the own body in a timing task within a perceptual-action loop will make a noticeable difference in timing performance, as compared to when humans only passively perceive the same perceptual scenario (Chen and Vroomen, 2013).

This Research Topic of “Sub- and supra-second timing: brain, learning and development” has integrated 16 submissions of novel research on sub- and supra-timing. We have categorized the papers in this topic into the following four themes, from which we can deduce trends of research about multisensory timing in the sub- and supra-second range.

## Sensory timing, interaction, and reliability

A central debate in sensory timing is whether it is subserved by a centralized timing mechanism or distinctive/modular processing (Ivry and Schlerf, 2008). We included five papers underlying this theme. Di Luca investigated how judgments of perceived duration are influenced by the properties of the signals that define the intervals. They found that timing distortion is attributed to both intervals (isochronous vs. an-isochronous) and filling types (empty vs. filled) (Horr and Di Luca). Cai and Eagleman asked themselves how the brain forms a representation of duration when each of two simultaneously presented stimuli influence perceived duration in different ways. They attributed the perceived averaged duration of simultaneously occurring visual stimuli to the weightings of the elementary (individual) stimuli, although the weighting performance did not fully predict statistically optimal integration. Birngruber et al. examined the effects of stimulus repetition vs. stimulus novelty on perceived duration. They substantiated the view that changesof simple, that is, semantically meaningless stimuli lead to shorter perceived duration of repeated as compared to novel stimuli. In the conceptual framework of the distinct timing hypothesis, Rammsayer et al. showed a gradual transition from a purely modality-specific, sensory-automatic to a more cognitive, a modal timing mechanism, by viewing the evidence that the prevalence of precision of auditory over visual timing disappeared when the temporal range is controlled. Yue et al. explored the effects of olfactory events upon reproduced time durations in auditory and visual modalities, and found that the biased timing in target stimuli (auditory and visual) could be accounted for by a framework of attentional deployment between the inducers (odors) and emotionally neutral stimuli (visual dots and sound beeps). However, the mechanisms of distinct timing vs. centralized timing are to be determined.

## Adaptive representation of time, learning, and temporal prediction

Golan and Zakay probed the duality of temporal encoding—the intrinsic and extrinsic representation of time—using fMRI. They exposed participants to stimuli with different temporal variance and found neural activation (within category-selective brain regions) increase as a function of increase in temporal variance. Thereafter, temporal encoding is an integral part of general perception. Moreover, time encoding on this level is an automatic process independent of attentional capacities. Tobin and Grondin compared expert and intermediate runners to compare their running time with their predicted time. Results show that task experience affects temporal prediction and accuracy in actual running time estimation in the order of many minutes. Zhang and Chen showed that time perception is adaptively recalibrated and biased by quick statistical binding of temporal information and non-temporal stimuli properties, by using a visual Ternus display as probe. Szelag et al. used temporal training protocols and explored the link between temporal information processing and language disorders (in aphasic patients and children with language impairment), and their therapy tools provide evidence for promising clinical applications.

## Sensorimotor synchronization, embodiment, and coordination

Under this topic, we included four studies. Booth and Elliott investigated individuals' ability to synchronize movements to a temporal-spatial visual cue in the presence of same modality temporal-spatial distractors and found early but not late visual distractors affect movement synchronization to a temporal-spatial visual cue. Hao et al. investigated the effect of voluntary movement on the simultaneous perception of auditory and tactile stimuli using a temporal order judgment task with voluntary movement, involuntary movement, and no movement, suggesting that the reference copy has a role in explaining the differential effects. In the framework of embodied time perception, Jia et al. showed that weight experience modulates visual duration estimation through the link between the weight of the backpack and the to be estimated visual target (backpack picture).

Osaka et al. extended the investigation of time perception to two agents and examined how two brains make one synchronized behavior using cooperated singing/humming between two people and hyperscanning–a new brain scanning technique. They found a significant increase in neural synchronization of the left inferior frontal cortex (IFC) as a neural signature for cooperative singing or humming.

## Perspective of psychological moment and temporal organization

The last part incorporates three papers which might provoke a re-thinking of concepts and methodology in sensory timing. Elliott and Giersch reconsidered the concept of “psychological moment” and suggested that within the 50–60 ms interval a more fine-scaled, serialized process structures and defines the passage of ongoing time. That is, a perceptual moment is experienced as co-temporality (two events are experienced as happening simultaneous) but on a level accessible through implicit behavioral measures nevertheless time is processed sequentially. Arstila discusses and then leans toward a brain time view, with respect to the debate of *time-marker view* vs. *brain time view*, a debate that is concerned with the question of how an observer extracts temporal information from a continuous stream of events. Zhou et al. propose that temporal aspects of objects can be treated as features of objects, and that psychological time or “apparent time,” similar to concepts underlying the analyses of reaction times, can serve as a tool to study the principles of neural codes related to object identity.

Overall, the collections in “Sub- and supra-second timing: brain, learning and development” show some recent trends and debates in multisensory timing research as well as provide a venue to inspire future work in multisensory timing.

## Author contributions

LC drafted the editorial, YB and MW revised it.

### Conflict of interest statement

The authors declare that the research was conducted in the absence of any commercial or financial relationships that could be construed as a potential conflict of interest.
